# Complete assembly of the *Leishmania donovani* (HU3 strain) genome and transcriptome annotation

**DOI:** 10.1038/s41598-019-42511-4

**Published:** 2019-04-16

**Authors:** Esther Camacho, Sandra González-de la Fuente, Alberto Rastrojo, Ramón Peiró-Pastor, Jose Carlos Solana, Laura Tabera, Francisco Gamarro, Fernando Carrasco-Ramiro, Jose M. Requena, Begoña Aguado

**Affiliations:** 1 Centro de Biología Molecular “Severo Ochoa” (CSIC/UAM), Campus de Excelencia Internacional (CEI) UAM+CSIC, Universidad Autónoma de Madrid, Madrid, Spain; 2 Instituto de Parasitología y Biomedicina “López-Neyra” (IPBLN-CSIC), Granada, Spain

## Abstract

*Leishmania donovani* is a unicellular parasite that causes visceral leishmaniasis, a fatal disease in humans. In this study, a complete assembly of the genome of *L*. *donovani* is provided. Apart from being the first published genome of this strain (HU3), this constitutes the best assembly for an *L*. *donovani* genome attained to date. The use of a combination of sequencing platforms enabled to assemble, without any sequence gap, the 36 chromosomes for this species. Additionally, based on this assembly and using RNA-seq reads derived from poly-A + RNA, the transcriptome for this species, not yet available, was delineated. Alternative SL addition sites and heterogeneity in the poly-A addition sites were commonly observed for most of the genes. After a complete annotation of the transcriptome, 2,410 novel transcripts were defined. Additionally, the relative expression for all transcripts present in the promastigote stage was determined. Events of *cis-*splicing have been documented to occur during the maturation of the transcripts derived from genes LDHU3_07.0430 and LDHU3_29.3990. The complete genome assembly and the availability of the gene models (including annotation of untranslated regions) are important pieces to understand how differential gene expression occurs in this pathogen, and to decipher phenotypic peculiarities like tissue tropism, clinical disease, and drug susceptibility.

## Introduction

Leishmaniasis is a group of diseases caused by protists of the genus *Leishmania*, which belong to the eukaryotic lineage Excavata and are classified within the order Trypanosomatida^1^. The list of pathogenic *Leishmania* species is large, even though the taxonomy of the genus is still under debate^2^. Nevertheless, some species are clearly linked to specific clinical syndromes^3^. Thus, *Leishmania major* most frequently causes cutaneous leishmaniasis (CL), which is characterized by the appearance of single to multiple skin ulcers, satellite lesions or nodular lymphangitis, but these affections more often resolve without treatment^4^. *Leishmania braziliensis*, which is exclusively distributed in South America, can cause mutilating mucocutaneous leishmaniasis (MCL). *Leishmania infantum* and *Leishmania donovani* are the causative species of the most severe form of disease, the visceral leishmaniasis (VL). According to recent estimates^5^, approximately 0.2 to 0.4 million VL cases and 0.7 to 1.2 million CL cases occur each year in the world. Unfortunately, there are no vaccines for human use and treatment of leishmaniasis relies on a small arsenal of drugs that, moreover, have important limiting factors in its use, like toxicity and emerging resistance^6^. Therefore, new developments, both vaccines and more effective drugs, are needed to control leishmaniasis. Better knowledge of the molecular biology of this parasite will help to achieve these objectives.

Most of the *Leishmania* species are digenetic, i.e. they need two hosts to complete their life cycle. *Leishmania* parasites are transmitted to vertebrate hosts by the bite of female phlebotomine sandflies; a remarkable association between sandflies and *Leishmania* species exists, being dictated by old relationships in evolutionary terms^7^. In the insect vector, the parasite survives and proliferates extracellularly in the alimentary tract; in contrast, in the vertebrate host, it adopts an obligatory intracellular form that thrives inside phagolysosomes^8^. The adaptation required to face these different host environments is achieved by modulating gene expression. However, *Leishmania* and related trypanosomatids are eukaryotes that possess unusual ways of controlling their gene expression^9^. Little, if any, regulation al the transcriptional level seems to exist; the genes, which are organized in large clusters with the same transcriptional orientation, but without any functional relationship, are constantly transcribed into long polycistronic precursor RNAs^10^. Following synthesis, the precursor transcripts are processed into mature mRNAs, each coding for an individual protein, by another unusual process known as *trans*-splicing^11^. The *trans*-splicing machinery, at specific positions, cuts the polycistronic precursor and a 39-nt long mini-exon (also named as spliced leader or SL) is added to the 5′-end of all mRNAs. In a coordinated manner, a poly-A tail is added to the 3′-end of mRNAs in such a way that polyadenylation of the upstream gene is directed by (and coupled to) the *trans*-splicing of the downstream gene^12^. The mini-exon contains a highly modified 5′-cap structure that, upon exportation of the mRNAs to the cytoplasm, is recognized by the translation machinery^13^. Although this polycistronic transcription might suggest that adjacent genes would have the same expression levels, it is known that this is not the case and strikingly different steady-state levels exist for collinear mRNAs^14^. Hence, mechanisms targeting post-transcriptional events, such as *trans*-splicing efficiency, mRNA nucleo-cytoplasmic transport, transcript degradation and translational efficacy are the relevant players controlling gene expression in *Leishmania*^9,15^. Ultimately, the fate of any given mRNA is determined by the ensemble of particular RNA-binding proteins (RBPs) that recognize specific sequences and/or structural motifs present in the mRNAs, mainly located at their untranslated regions (UTRs)^16,17^. In this context, physical delimitation of UTRs and definition of accurate gene models are paramount for deciphering the regulatory networks that control gene expression not only in *Leishmania* but in any eukaryotic cell^18^.

From a genomic approach, the basic step for establishing gene models is the determination of the complete genome sequence of an organism. The first sequenced genome for a *Leishmania* species was that of *L*. *major* (Friedlin strain); this was attained after a hard experimental labour, following meticulous strategies and involving large dedicated sequencing centres^19^. This genome assembly remains as one of the more robust *Leishmania* genomes determined to date, and, since then, only few and small modifications have been introduced^20^. During the last decade, the extraordinary progress in sequencing due to the development of the so-called next-generation sequencing (NGS) technologies together with a significant reduction of sequencing costs have enabled the determination of genome sequences for many *Leishmania* species and strains^21^. However, even though this information is really valuable, the quality of genome assemblies is lower than that achieved in 2005 for the *L*. *major* (Friedlin strain) genome. Nevertheless, in a recent work, by the combination of sequencing data derived from two NGS- platforms, the Pacific Biosciences (PacBio) technology, which produces long sequencing reads, and the Illumina technology, which yields shorter but more accurate sequences, a complete assembly for the *L*. *infantum* (JPCM5 strain) was obtained^22^.

On the other hand, the establishment of gene models requires not only the prediction of open reading frames (ORFs) but also the delimitation of UTRs, which are regions earmarked to govern the fate of the mRNA molecules inside the cell^23–25^. In fact, the *Leishmania* genome contains a remarkable number of genes sharing identical ORFs but differing substantially in their UTRs^26–32^. Moreover, several studies dealing with expression levels of particular genes in *Leishmania* have evidenced the relevance of the 5′- and/or 3′- UTRs in mediating differential transcript abundances and translation efficacies along the parasite life cycle^33–37^. For a long time, identifying the full set of transcripts present in a cell or organism, i.e. the transcriptome, was envisioned as impractical given that this task required the sequencing of large numbers of expressed sequence tag (EST) sequences, an approach that otherwise is biased for the abundance of the different RNA molecules. Again, in this research field, the application of NGS technologies for RNA sequencing (RNA-seq) has allowed to reveal the landscape and dynamics of particular transcriptomes with unprecedented level of depth and accuracy^38^. However, to date, there are few studies aimed to deciphering the transcriptomes in *Leishmania*^39^. *L*. *major* was the first species of the genus *Leishmania* in which a complete poly-A^+^ transcriptome was generated; a total of 10,285 transcripts were identified, 1,884 of which did not correspond to previously predicted genes and 410 miss-annotated ORFs were corrected^14^. *L*. *mexicana* was the second *Leishmania* species to have annotated its transcriptome, consisting of 9,169 transcripts matching with previously predicted ORFs and 936 novel transcripts^40^. Additionally, the *L*. *major* repertoire of snoRNAs was determined by affinity purification of the SNU13 and NHP2 RNPs and RNA-seq analysis; the study identified 81H/ACA and 80 C/D snoRNAs^41^.

Here, we describe the first complete assembly for the *L*. *donovani* genome, and this information, combined with RNA sequencing (RNA-seq), was used to generate a comprehensive transcriptome. Thus, our study is providing the gene models for this *Leishmania* species that will guide further investigations into the molecular mechanisms responsible for differential gene expression along its life cycle and in response to environmental stresses such as drug treatment.

## Materials and Methods

### Leishmania culture

Promastigotes of *L*. *donovani* (MHOM/ET/67/HU3) were grown at 26 °C in RPMI 1640-modified medium^42^. In detail, RPMI 1640 (Gibco, Ref 51800-043) medium was supplemented with 13.3 mM glutamine, 2.5 mM arginine, 0.3 mM cystine,1.7 mM glutamate, 62.1 mM proline, 0.6 mM ornithine, 3.8 mM glucose, 2.2 mM fructose, 5.1 mM malate, 2.8 mM α-ketoglutarate, 0.5 mM fumarate, 0.5 mM succinate, 25 mM Hepes, 50 µg/ml gentamicin, 2 × MEM vitamins (Gibco); after adjusting pH to 7.2, heat-inactivated foetal bovine serum (HIFBS, Gibco) was added to a final concentration of 20%. Alternatively, in the experiments dealing with the isolation of DNA for PacBio sequencing, promastigotes of the same strain were cultured at 26 °C in M199 medium (Sigma-Aldrich) supplemented with 10% HIFBS (Biowest), 40 mM Hepes (pH 7.4), 0.1 mM adenine, 10 µg/ml hemin, 1 µg/ml biotin, 2 ng/ml biopterin, 100 U/ml penicillin G and 0.1 mg/mL streptomycin sulphate.

### DNA and RNA isolation

RNA was prepared from around 4 × 10^8^ promastigotes in the late logarithmic phase; after harvesting by centrifugation, the pellet was suspended in 1 ml of TRI Reagent (Sigma-Aldrich, product No. T9424). Manufacturer’s instructions were followed. Samples were kept at −70 °C for a week before proceeding with the phase separation. After thawing, 0.2 ml of chloroform was added, and the mixtures were shaken vigorously for 15 sec. After centrifugation, three phases were observed: a red organic phase (containing protein), an interphase (containing DNA), and a colorless upper aqueous phase (containing RNA). Both interphase and aqueous phases were processed separately for isolation of DNA and RNA, respectively. RNA samples were suspended in DEPC-treated water, and their concentrations were determined using the Nanodrop ND-1000 (Thermo Scientific); all samples showed A_260_/A_280_ ratios higher than 2.0. In addition, RNA integrity was checked in a bioanalyzer (Agilent 2100). The DNA samples were also quantified by absorbance at 260 nm using the Nanodrop, and the integrity analyzed by agarose gel electrophoresis. For PacBio sequencing, DNA was prepared following a classical phenol extraction method^43^.

### Illumina sequencing of DNA and reads assembly

Library construction and paired-end library sequencing were performed at the Centro Nacional de Análisis Genómico (CNAG-CRG, Spain) using Illumina HiSeq. 2000 technology. A total of 16,980,871 paired-end, 101 bp sequence reads were generated. PrinseqQuality (http://prinseq.sourceforge.net/) was applied to quality filtering/trimming of reads (cut-off value, 20), and only reads with length ≥60-nt were used. Reads were assembled using the CLC Genomics Workbench version 5.0 (CLC Bio).

### PacBio sequencing and de novo assembly

The single-molecule real-time (SMRT) sequencing technology developed by Pacific Biosciences (PacBio) was used for long-read sequencing. A total of 312,388 pre-filtered reads were generated on a PacBio RS II sequencing instrument. The sequencing service was provided by the Norwegian Sequencing Centre (www.sequencing.uio.no), a national technology platform hosted by the University of Oslo and supported by the “Functional Genomics” and “Infrastructure” programs of the Research Council of Norway and the South-Eastern Regional Health Authorities. Quality trimming of PacBio reads was done by default parameters as part of the HGAP pipeline (P_filter Module).

*De novo* genome assembly was carried out following a hierarchical genome-assembly process (HGAP^44^), using the HGAP v3 (PacBio, SMRT Analysis Software v2.3.0) and HGAP4 (PacBio, SMRT Link 4.0.0) protocols. Three different assemblies were performed with HGAP by varying the size of the expected genome (34 and 35 Mbp for HGAP3, and 35 Mbp for HGAP4).

### Assembly refinements

The contigs, initially assembled by HGAP (varying the GenomeSize parameter) from the PacBio reads, were checked in order to discard those having a disproportionately low coverage (<40x) or short length (<15-Kb). To assign the correspondence between contigs and chromosomes, BLAST searches^45^ were performed between the assembled contigs and current *L*. *major* (Friedlin strain) genome^19^. Finally, a total of 41 contigs were determined as *bona fide* genomic sequences. Thirty-one of these contigs were found to correspond to complete chromosomes. To complete assemble the other five chromosomes, their corresponding contigs were joined using minimus2 pipeline^46^. Firstly, the gap size between contigs was calculated (lower than 5-kb in all cases) and, based on the Illumina reads, the gaps were closed by Gapfiller^47^, which takes into account the mean size of the paired-end reads.

On the other hand, the contigs generated from the Illumina sequencing reads (see above) were aligned to the PacBio reads-based assembly using LAST aligner (http://last.cbrc.jp/). This allowed the identification of Illumina contigs that aligned with the chromosomal ends and therefore to extend the chromosomes. Several tools were used to accurately extend the chromosomal ends. Thus, for chromosomes 7, 14, 31 and 36, the optimal extension was attained with MAFFT multiple-aligner software^48^. For chromosomes 10, 13, and 35, the best extension was obtained by BLAST alignment. Additionally, for the rest of chromosomes, the SSPACE-standard software^49^ was used.

Finally, sequence corrections were performed in the draft assembly using PacBio-utilities (indel-targets and indel-apply tools; https://github.com/douglasgscofield/PacBio-utilities). This tool uses paired end Illumina reads and it is designed for detecting single-base deletions introduced with low frequency in homopolymer strings by the PacBio platform. Sequence insertions/deletions (indels) were introduced when they were supported by more than 10 Illumina reads and the insertion/deletion was present in 80% (or above) of the reads mapping the concerned position. Furthermore, an in-house Python script, which uses the results provided by Pilon tool^50^, was designed to assign the position when ambiguous indels were detected. A total of 3,098 indels were corrected, of which 3,061 corresponded to sequence insertions and 37 to deletions.

### Alignments and coverage maps

A coverage analysis on the newly assembled chromosomes was performed using both Illumina and PacBio reads. Illumina reads were aligned by Bowtie2^51^ and PacBio bax.h5 reads were aligned by pbalign (which uses the BLASR method^52^). Coverage analysis was done from each alignment along the 36 chromosomes using the GenomeCoverageBed tool^53^. Coverage data were smoothed using an in-house Perl script that calculates the mean coverage over a one bp step sliding window with a size of 200 bp. The coverage plots were generated using GNUPLOT (http://www.gnuplot.info/).

### SNP identification

Firstly, the BWA-MEM tool^54^ was used to align the Illumina reads to the *L*. *donovani* (HU3) genome. Afterwards, the picard tool (http://broadinstitute.github.io/picard/) was used to remove read duplicates. The selected reads were realigned to the genome by GATK (version 3.7; www.broadinstitute.org/gatk/). Finally, variant calling was done by two methods, the GATK HaplotypeCaller v. 3.7^55^ and the Freebayes version 1.1.0^56^. In both methods, the quality filters (depth above 9 and quality above 10) were applied.

### Annotation of protein-coding genes and known non-coding RNAs

Bulk annotation of the assembled *L*. *donovani* HU3 genome was performed using Companion web server^57^ with the default settings, and selecting the *L*. *major* (Friedlin strain) annotation as a reference genome. OrthoMCL^58^ and BLAST searches were performed to establish orthology between *L*. *donovani* (HU3) and *L*. *major* (Friedlin) genes. All this information was combined into a GFF3 file using an in-house script written in Python. The automatic ID codes generated by Companion were accommodated to the transcript nomenclature (see below, transcriptome annotation section), and the annotated genes were named with the label LDHU3_XX.YYYY, where XX identifies the chromosome number and YYYY is the serial number assigned to the transcript in which the ORF is found. For structural RNAs, it is indicated the RNA type, the chromosomal location and a serial number of three digits (e.g. LDHU3_TRNA.02.001).

### Transcriptome annotation

A total of 56,506,556 (2 × 76-nt) stranded RNA-seq reads, derived from three biological replicates, were generated using the Illumina HiSeq. 2000 technology (CNAG-CRG, Spain) as described elsewhere^59^. The *L*. *donovani* (HU3) transcriptome was generated following the pipeline described by Rastrojo *et al*.^14^. In brief, after going through the standard Illumina quality-filtered process, the reads were further analysed using FASTQC to assure adequate quality. RNA-seq reads were then mapped to the *L*. *donovani de novo* genome (generated in this work) using Bowtie2 aligner with default parameters. Lastly, mapped reads were assembled into transcripts using Cufflinks^60^ with default parameters. On the other hand, among the unaligned reads, a search was performed, using a Perl in-house script, looking for reads that contained eight or more nucleotides identical to the 3′-end of the SL sequence (AACTAACGCTATATAAGTATCAGTTTCTGTACTTTATTG). After removing the SL-derived nucleotides, the trimmed reads were mapped back to the *L*. *donovani* (HU3) to define the position of the corresponding SL-addition site (SAS). For the identification of poly-A addition sites (PAS), a similar procedure was performed. Here, the presence of an A-string longer than 5 nucleotides at the end of the reads was used to uncover potential PAS. A Perl script was developed to trim the transcripts generated by Cufflinks according to the positions of the mapped SAS and PAS. Finally, the transcripts were visualized and manually curated (if required) using the Integrative Genomics Viewer (IGV^61^). During this revision, some previously annotated ORFs had to be corrected because they started before the beginning of the transcript. For transcript nomenclature, a serial number (four-digits), according to its chromosomal location (from left to right) and increasing by 10, was added after the chromosome ID and labelled with the word ‘T’, e.g. LDHU3_01.T0010 (i.e. first transcript annotated on chromosome 1).

### Determination of RNA levels from RNA-seq data

RNA-seq data were obtained from three different cultures of *L*. *donovani* HU3 promastigotes in order to determine relative levels of every transcript in the transcriptome. Transcript levels were expressed as FPKM (fragments per kilobase of transcript per million mapped reads). This parameter reflects the abundance of a transcript in the sample by normalizing for RNA length and the total read number^62^. In this study, Cufflinks tool^60^ was used to calculate the FPKM.

## Results and Discussion

### De novo assembly of the *L*. *donovani* (HU3 strain) genome

**Figure 1:**
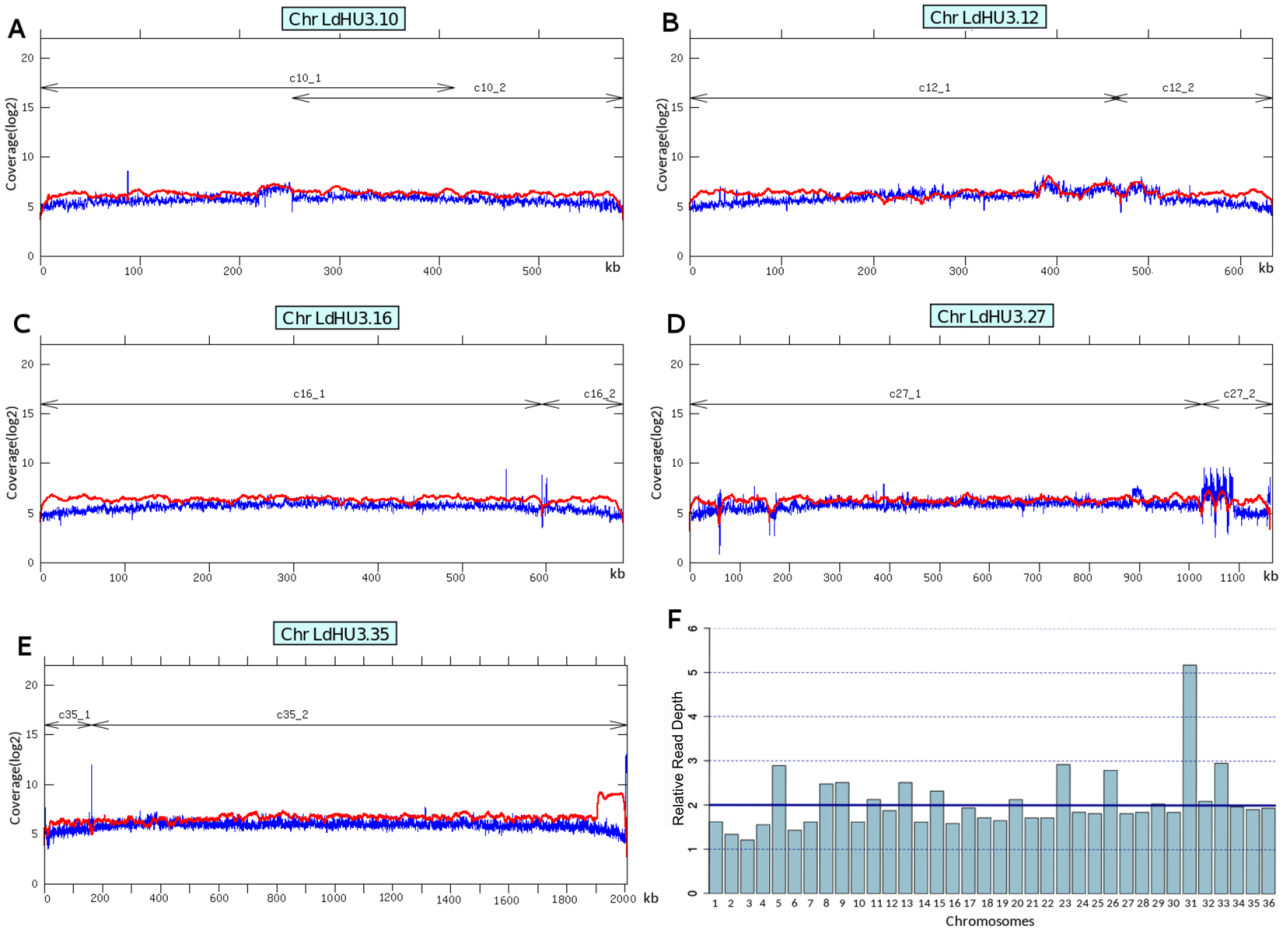
Read-depth analyses along the chromosomes generated by the fusion of two PacBio-assembled contigs (panels A–E). Coverage (log2 scale) was determined by sliding window analysis (bin 200 bp) with either Illumina (in blue) or PacBio (in red) reads, along chromosomes 10, 12, 16, 27 and 35. The size and position of the contigs used are shown by lines with arrow-heads. Panel F, relative somy of the *L*. *donovani* (HU3) chromosomes. The somy estimation was performed using a 2-loop method^77^. The median coverage of the genome is shown by a solid line, and it was assigned as 2, taking into account that diploid is considered the major ploidy status in *Leishmania*. The dotted lines indicated the estimated values for other somies. Graphs were generated from the median coverage values for each chromosome using the barplot function of R package (https://cran.r-project.org).

The *L*. *donovani* HU3 strain (MHOM/ET/67/HU3), also known as LV9 or L82, is one of the most widely used strains in studies regarding drug resistance and other molecular aspects^63–65^. It is a cloned line, as reported elsewhere^66^. Firstly, DNA from LdHU3 promastigotes was sequenced using Illumina technology, obtaining 16,980,871 (2 × 101 bp) reads with a median insert size of 295-bp. Taking into account the estimated size (32,4-Mb) for the *L*. *donovani* genome assembly reported by Downing *et al*.^67^, these reads would account for an average sequencing depth of 105x. Several assemblers were tried in order to get a *de novo* assembly, obtaining the best results with CLC Genomics Workbench software (CLC Bio; version 5.0). However, the attained assembly represented a fragmented genome composed by 2,545 contigs and 1,224 gaps that amounted for a total genome size of 30,221,623 bp, being the longest contig of 201,094-bp. *Leishmania* genomes are rich in repeated sequences (0.4–1 kb in length) that are scattered along the different chromosomes^68,69^, and this is a cause of conflict for assemblers that work with short reads as those generated by Illumina platforms^70,71^. In order to improve the genome assembly, we also obtained long-read sequences, generated by the PacBio single-molecule real-time (SMRT) sequencing technology^72^. A total of 312,388 reads, with an average length of 11,900-bp were obtained. According to the genome size of the *L*. *donovani* BPK strain (Table 1), these figures would account for an 89× mean coverage. Following the methodology detailed in the Materials and Methods section, a *de novo* assembly resulted in the generation of 85 contigs, even though 44 of them were discarded because of their small size and the fact that they were supported by a low number of reads (spurious contigs). Afterwards, using several assemblers and other bioinformatics tools, together with the contigs generated from Illumina-reads (see Assembly refinements in Methods section), it was possible to join some of the 41 contigs to a final number of 36 contigs, matching the number of chromosomes existing in this *Leishmania* species^73^. Chromosomes 10, 12, 16, 27 and 35 resulted from the joining of two PacBio contigs (Fig. 1), whereas the rest of chromosomes corresponded directly to individual PacBio contigs. As shown in Fig. 1, a continuous and homogenous distribution of reads was observed after the alignments of both types of sequences (Illumina and PacBio reads) against the assembled chromosomes; this finding supports that a correct assembly was attained.

**Table 1:** Features of the *L*. *donovani* assembled genomes and sequencing strategies used.

Features	Strain BPK282A1^a^	LD-974^b^	HU3^c^
Chromosomes (scaffolds)	36	36	36
Number of contigs	2154	1100	36
Annotated genes	8195	8474	8595
Annotated CDS	8070	8336^d^	8405
Annotated pseudogenes	13	NA^e^	47
Annotated structural genes	112	138	143
Number of gaps	2118	1064	0
Number of Ns	1192833	NA	0
Haploide genome size (bp)	31252135	27848322	33035865
Illumina coverage median	52	~110	105
454 GS FLX Titanium coverage median	22	—	—
PacBio coverage median	—	—	89

^a^See ref.^67^.

^b^See ref.^92^.

^c^This work.

^d^Predicted, but non-annotated.

^e^Not available.

A singularity in the coverage was observed in the right end of chromosome 35 (Fig. 1E), the read depth of PacBio reads in the 3′ end (around 100-kb) was clearly higher than that observed in the rest of the chromosome. It was postulated the existence of an extrachromosomal amplicon covering this region. We analyzed whether this region may be related to the LD1 extrachromosomal amplicon, which has been observed very often in *L*. *donovani* and other *Leishmania* species^63^. BLAST analysis, using a partial sequence (7.1-kb) of the *L*. *infantum* LD1 amplicon determined by Myler *et al*.^74^, showed a 99% of sequence identity with the region 1,904,625-1,911,724 of the *L*. *donovani* chr35; interestingly, this region marks the point in which the PacBio coverage increases. Nevertheless, the Illumina coverage did not show a similar increase. A plausible explanation to this fact may be found in the different growth media used for culturing *L*. *donovani* promastigotes before DNA isolation for each one of the sequencing methodologies (see Material and Methods for further details). In brief, DNA for Illumina sequencing was isolated from promastigotes cultured in RPMI medium supplemented with 20% foetal bovine serum (FBS) and DNA isolation for PacBio sequencing was independently obtained from promastigotes of the same HU3 strain but cultured in M199 medium supplemented with 10% FBS. Our hypothesis is that the LD1 amplicon was generated during the culturing in M199 medium.

Table 1 summarizes the main features of the assembled genome for the strain HU3, generated in this work, and its comparison with the two other *L*. *donovani* assemblies published to date. This new assembly represents an improved genome regarding the current reference genome (BPK strain^67^), which is incomplete and presents some annotation deficiencies^75^. Thus, apart from eliminating the gaps, the genome size has been extended about 1.8 Mb in length and the total number of annotated genes has been increased by 400 genes, regarding the current reference *L*. *donovani* (BKP strain) genome. Additionally, after comparing the HU3 assembled genome and the BKP one, significant reorganizations in ten chromosomes were observed (see Figs S1 to S10 in Supplementary Information File). However, it is likely that these differences in chromosomal structure between both strains may be rather apparent than real, taking into account the fragmented assembly, currently available for the BKP strain. This question might be addressed when a full assembled genome for this strain is generated.

The assembly of the *L*. *donovani* HU3 genome cannot be considered as the final one, mainly considering the chromosomal extremities. Thus, telomeric TAGGGT repeats were found at the ends of many chromosomes, but not in all. They were found at the 5′ ends of the chromosomes 1, 5, 8, 14, 15, 16, 18, 22, 24, 27, 28, 32, 33 and 35, and at 3′ ends of the chromosomes 4, 7, 9, 12, 13, 15, 18, 20, 23, 24, 26, 27, 28, 29, 31, 32, 34 and 35. Chiurillo and co-workers^76^ have documented the existence in all *L*. *donovani* chromosomes of complex arrays of repeated and non-repeated sequences, adjacent to the telomeric repeats. It is likely that the complexity of these arrays and the intrinsic difficulty of sequencing the chromosomal ends have precluded the complete assembly of the extremities for all the *L*. *donovani* chromosomes.

Based on the Illumina read coverage, the somy for each chromosome was estimated by the 2-loop method^77^. The results indicated that most of the chromosomes in this strain are diploid (Fig. 1E). Chromosomes 5, 23, 26 and 33 would be trisomic and chromosome 31 appeared as pentasomic. The somy status is similar to that determined for other *L*. *donovani* strains^67,78,79^.

Additionally, we analyzed the degree of allelic heterozygosity by using two SNP-calling predictors, GATK HaplotypeCaller v. 3.7 and Freebayes version 1.1.0 (see Materials and Methods for further details). The results were very similar, GATK identified 4,622 SNPs, and 4,865 SNPs were annotated by Freebayes. Rogers *et al*.^78^, using also Illumina reads to call heterozygous SNPs in the reference genomes of *L*. *major*, *L*. *infantum*, *L*. *braziliensis* and *L*. *mexicana*, identified 297, 629, 44,588 and 12,531 SNPs, respectively. However, it is likely that the degree of heterozygosity may be a strain-specific trait rather than a species-specific one.

Table 2 shows the sizes and the number of annotated genes for each one of the 36 chromosomes that comprise the genome of this *Leishmania* species. The genome size and the number of annotated genes coding for proteins are very similar to those determined in other *Leishmania* species for which their genomes have been completely assembled. Thus, the *L*. *major* (Friedlin strain) genome has a size of 32,816,678 bp and 8,272 protein-coding genes were annotated^19^. The size of the *L*. *infantum* (JPCM5 strain) genome is 32,802,969 bp, and 8,645 protein-coding genes were annotated^22^. The size for the *L*. *donovani* (HU3 strain) genome assembled in this work was 33,035,865 bp, and the number of protein-coding genes was 8,405 (Table 1).

**Table 2:** Chromosomal sizes and number of annotated genes in the *L*. *donovani* (HU3 strain) genome.

Chromosome	Size (bp)	Annotated genes	Chromosome	Size (bp)	Annotated genes
**1**	292351	85	**19**	718213	176
**2**	361533	73	**20**	741387	175
**3**	387516	99	**21**	764502	232
**4**	475442	128	**22**	740689	172
**5**	467653	154	**23**	785035	211
**6**	521439	138	**24**	860435	247
**7**	591743	134	**25**	897487	264
**8**	563529	135	**26**	1062302	277
**9**	574290	180	**27**	1162458	280
**10**	584800	153	**28**	1177572	327
**11**	601398	147	**29**	1263082	310
**12**	635222	130	**30**	1402142	389
**13**	649940	169	**31**	1543155	358
**14**	650104	160	**32**	1564662	422
**15**	658046	164	**33**	1553627	377
**16**	691230	178	**34**	1895418	485
**17**	701758	182	**35**	2008222	540
**18**	718943	172	**36**	2768540	772
			**Genome**	**33035865**	**8595**

After sequencing the *L*. *braziliensis* and *L*. *infantum* genomes^80^, and their comparison with the *L*. *major* genome, it was surprising to realize the quite small number of species-specific genes existing in the three *Leishmania* species, taking into account both the large evolutionary distance that separates those species and the different pathologies they produce^81^. In particular, only five *L*. *major*–specific genes and 26 *L*. *infantum*–specific genes were identified^80^. Hence, we considered of interest to determine the number of species-specific genes existing in *L*. *donovani* by comparing them with the *L*. *major* annotated genes. *L*. *donovani* and *L*. *infantum* species are genetically almost indistinguishable^82^ and both cause VL in humans, whereas *L*. *major* causes cutaneous affections and diverged from the *L*. *donovani*/*L*. *infantum* complex parasites more than 10 million years ago^7^. Gene annotation of the assembled *L*. *donovani* HU3 genome was performed using the Companion web server^57^, selecting the *L*. *major* (Friedlin strain) annotation as a reference. An initial analysis indicated that 153 of the protein-coding genes annotated in the *L*. *donovani* genome did not have orthologues in the *L*. *major* database. However, an individual analysis of these predicted genes allowed us to determine that 97 out of the 153 protein-coding genes are present, but currently non-annotated, in the *L*. *major* genome. Another 41 *L*. *donovani* annotated genes showed remarkable sequence identity with *L*. *major* genomic regions, but the existence of stop codons suggested that these must be pseudogenes in *L*. *major*. Finally, for the remaining 15 *L*. *donovani* annotated genes (see Table 3), no significant sequence homology was found and, therefore, they can be considered as genes lacking orthologues in *L*. *major*.

**Table 3:** *L*. *donovani* annotated genes without orthologues in the *L*. *major* genome.

LDHU3.03.0460	hypothetical protein
LDHU3.03.0900	CDP-alcohol phosphatidyltransferase, putative
LDHU3.08.0620	cyclopropane-fatty-acyl-phospholipid synthase
LDHU3.23.1360	hypothetical protein
LDHU3.28.3370	hypothetical protein
LDHU3.29.1980	hypothetical protein
LDHU3.29.2050	Amastin surface glycoprotein, putative
LDHU3.29.2080	Amastin surface glycoprotein, putative
LDHU3.29.2100	tuzin like protein, putative
LDHU3.31.2550	hypothetical protein
LDHU3.32.3250	hypothetical protein
LDHU3.33.4860	Zn-finger in Ran binding protein, putative
LDHU3.34.4290	hypothetical protein
LDHU3.36.0800	sec. 14, cytosolic factor
LDHU3.36.5620	Nucleotidyltransferase domain containing protein, putative

The 15 LdHU3-specific CDSs (Table 3) are present in both the *L*. *donovani* BPK genome^67^ and the *L*. *infantum* genome^22^. Nevertheless, three of them are truncated in the BPK reference genome and seven were not annotated. In contrast, all of them are present in the *L*. *infantum* genome and 13 of them are currently annotated.

More pronounced differences between *L*. *major* and *L*. *donovani* genomes exist when pseudogenes are considered. Thus, among the *L*. *donovani* annotated genes, 47 were classified as pseudogenes based on either the presence of internal stop codons or incomplete ORF (Supplementary File 1, Table S1). Remarkably, except for one pseudogene that is also annotated as pseudogenic (LmjF.23.0910), the orthologues to the *L*. *donovani* pseudogenes seem to be functional genes in the *L*. *major* genome. Conversely, 38 out of the 39 pseudogenes currently annotated in the *L*. *major* genome^19^ seem to be functional genes in *L*. *donovani*. Therefore, it is likely that some phenotypical and virulence traits specific for each species can arise from differences in the degree of ‘pseudogenization’ of particular genes.

### *L*. *donovani* (HU3 strain) genome contains many tandemly repeated genes

**Figure 2:**
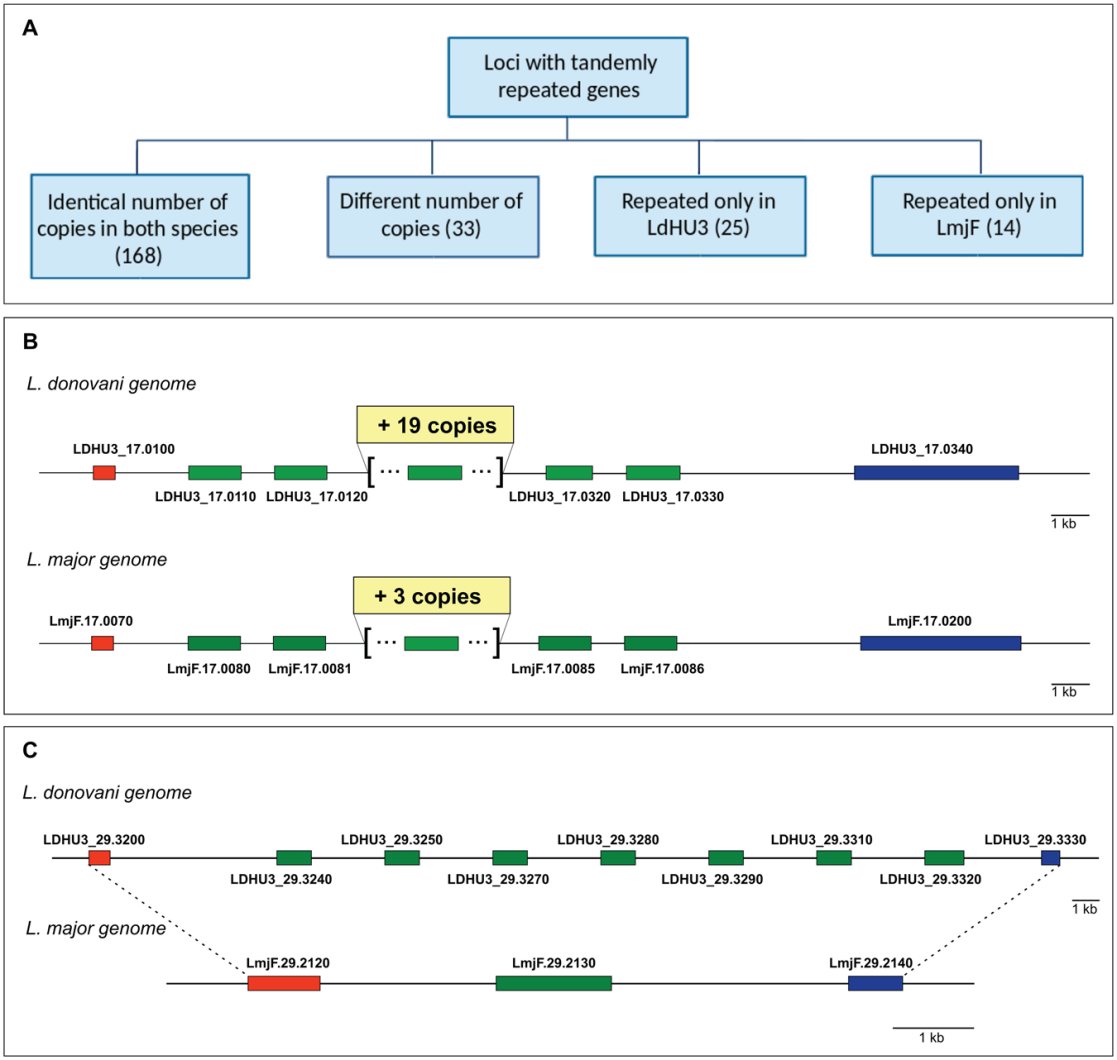
Comparison of tandemly repeated genes existing in the *L*. *donovani* (LdHU3) and the *L*. *major* (LmjF) genomes. Panel A, tandemly repeated genes in both species distributed according to the similarities/differences in the number of copies. Panel B, schematic representation of the loci coding for the elongation factor 1-alpha genes in the *L*. *donovani* and *L*. *major* genomes. The repeated copies are shown as green boxes in both species. The flanking ORFs are shown in red (ADP-ribosylation factor-like protein 1) and dark blue (receptor-type adenylate cyclase A). Panel C, genes encoding for a hypothetical protein (with a leucine-rich repeat domain) are shown as green boxes in both species. The flanking ORFs are shown in red (VIT family, putative) and dark blue (C-8 sterol isomerase-like protein).

*L*. *major* (Friedlin strain) genome is considered the ‘gold standard’ among the *Leishmania* genome assemblies because it was the first to be sequenced^19^ and, surely, the best assembled until recently^22^. A synteny analysis comparing the *L*. *major* (Friedlin) and the *L*. *donovani* (HU3 strain) genomes indicated an extremely conservation of gene order, even though a few genomic reorganizations were observed (data not shown). However, significant differences regarding the number of copies in loci with tandemly repeated genes exist between both species (Fig. 2A). A total of 240 loci with tandemly repeated genes were identified, and the majority of the loci (168; listed in Supplementary File 1, Table S2) were found to contain identical number of copies in both species. In addition, 33 loci with tandemly repeated genes exist in both species, but the number of genes is different for each species (those loci are listed in Supplementary File 1, Table S3). An example is shown in Fig. 2B, the elongation factor1-alpha locus is composed by 23 tandemly linked genes in *L*. *donovani* (HU3 strain) whereas only seven copies were assembled in the *L*. *major* (Friedlin) genome. Finally, *L*. *donovani* (HU3) genome has 25 loci with tandemly repeated genes that are single copy in *L*. *major* (Friedlin), and the *L*. *major* genome contains 14 tandemly repeated loci that in *L*. *donovani* have only a gene (see Supplementary File 1, Tables S4 and S5, respectively, for lists of these loci). As an example, Fig. 2C illustrates the case of LDHU3_29.3240-3320 locus, coding for a hypothetical protein having a leucine-rich repeat domain, that is composed by seven genes in *L*. *donovani* but a sole gene copy (LmjF.29.2130) in the *L*. *major* genome. Several authors have proposed that differences in gene copy number may contribute to modulate gene expression levels, providing additional genetic contributions to species-specific differences in parasite tropism and disease outcome^78,83^.

### Transcriptome of *L*. *donovani* HU3 strain

**Figure 3:**
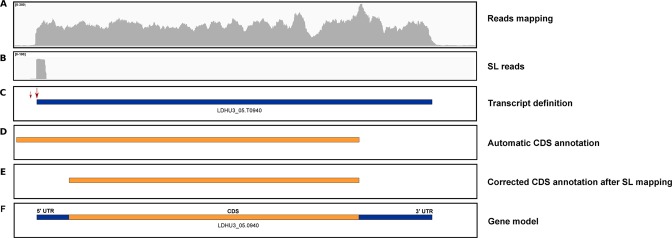
Illustration of the process followed for correcting the automatic annotation of CDS based on the transcript definition. Panel A, mapping of RNA-seq reads in the genomic region expanding the LDHU3_05.0940 gene. Panel B, mapping of SL-containing RNA-seq reads (two SL-addition sites (SASs) were mapped, the main site was covered by 72 reads and the secondary –small arrow- by only 2). Panel C, LDHU3_05.T0940 transcript annotation based on the position of the main SAS and the polyadenylation site (not shown). Panel D, automatic CDS annotation generated by Companion. Panel E, manually corrected CDS annotation, after delimiting the transcript. Panel F, proposed gene model for the LDHU3_05.0940 gene.

After the assembly of the genome for this *L*. *donovani* strain, the definition of the poly-A+ transcriptome was undertaken (see Methods section for procedure details). Finally, 10,893 transcripts/gene models were defined (see Supplementary file 2 for the complete list), of which 8,452 corresponded to genes with annotated protein-coding sequences (CDS), whereas the remaining 2,441 lacked annotated CDS. In the latter group, SL addition sites (SAS) were observed in most of them (2,301, 94%), even many of them presented two or more alternative SAS (1,781, 73%). Regarding those annotated as protein-coding transcripts, SAS were identified for nearly all the transcripts (8430, 99.7%) and two or more alternative SAS were defined for 7,792 (92.2%) of them. Moreover, 1,565 transcripts contained alternative SAS within the predicted ORF. In fact, for 249 genes, the ORF predicted by bioinformatics tools had to be corrected during the annotation of the *L*. *donovani* HU3 genome (above) due to the finding that transcripts were shorter than the predicted ORF, and no alternative SAS were found outside the ORF. In those cases, the ORF predicted by bioinformatics means had to be re-annotated taking into account the first, in-phase ATG initiation codon, located within the delineated transcript. An example of such events is shown in Fig. 3. Thus, the transcript LDHU3_05.T0940, delimited by SL and polyadenylation addition sites and supported by the RNA-seq coverage (panel A–C), was found to start downstream of the automatic CDS annotation, done by bioinformatics tools (panel D). Therefore, a new ORF had to be defined within the transcript sequence (panel E). Finally, after combining all this information, a plausible gene model (i.e. gene LDHU3_05.0940) was generated (panel F).

Among the 2,441 transcripts lacking CDS annotation, 31 contained structural RNAs and the rest were categorized as novel transcripts. CDS annotation was done using Companion server, and this tool was set to predict new putative polypeptides from ORF larger than 200 bp. However, an ever growing number of short CDS-encoded peptides are being characterized and found to play physiological functions in metazoans, plants and in unicellular organisms^84,85^. Therefore, we must be cautious in considering that most of these novel transcripts are non-coding RNAs (ncRNAs). Transcripts lacking gene-annotation were also identified during the transcriptome annotation of two other *Leishmania* species, *L*. *major*^14^ and *L*. *mexicana*^40^. In order to know the degree of conservation of these novel transcripts among the three species, a BLASTn analysis was done using these three groups of novel transcripts and the results are summarized in Table 4. Thus, 1,513 out of the 2,410 novel transcripts annotated in this work for *L*. *donovani* can be categorized as homologues to novel transcripts annotated in *L*. *major*. The number of *L*. *donovani* novel transcripts with homologues in the *L*. *mexicana* was 899, but it may bear in mind that only 936 novel transcripts were annotated in the *L*. *mexicana* transcriptome^40^. It is worth noting that more restricted criteria were used to define a “novel transcript” in the *L*. *mexicana* transcriptome analysis than in the other two analyses (*L*. *major* and *L*. *donovani*); in the former, only transcripts having an ORF longer than >75 nucleotides were categorized as novel transcripts^40^. Nevertheless, this analysis showed the existence of a significant number of species-specific transcripts (Table 4), whose functional roles need to be addressed in future works.

**Table 4:** Analysis of the conservation of the novel transcripts identified in the *L*. *donovani* (LdHU3), *L*. *major* (LmjF) and *L*. *mexicana* (LmxM) transcriptomes.

Transcripts	LdHU3^a^	LmjF^b^	LmxM^c^
Total novel transcripts	2410	2143	936
Homologs in the LdHU3 transcriptome	—	1349	590
Homologs in the LmjF transcriptome	1513	—	598
Homologs in the LmxM transcriptome	893	762	—
Conserved in the three species	770	617	444
Species specific transcripts	773	647	192

^a^This work.

^b^LmjF_cbm_v1_MT_v1.2.gtf transcriptome available in Leish-ESP Web server (http://leish-esp.cbm.uam.es/L_major_downloads.html).

^c^See ref.^40^.

### The transcript coding for poly(A) polymerase is also processed by cis-splicing in *L*. *donovani*

**Figure 4:**
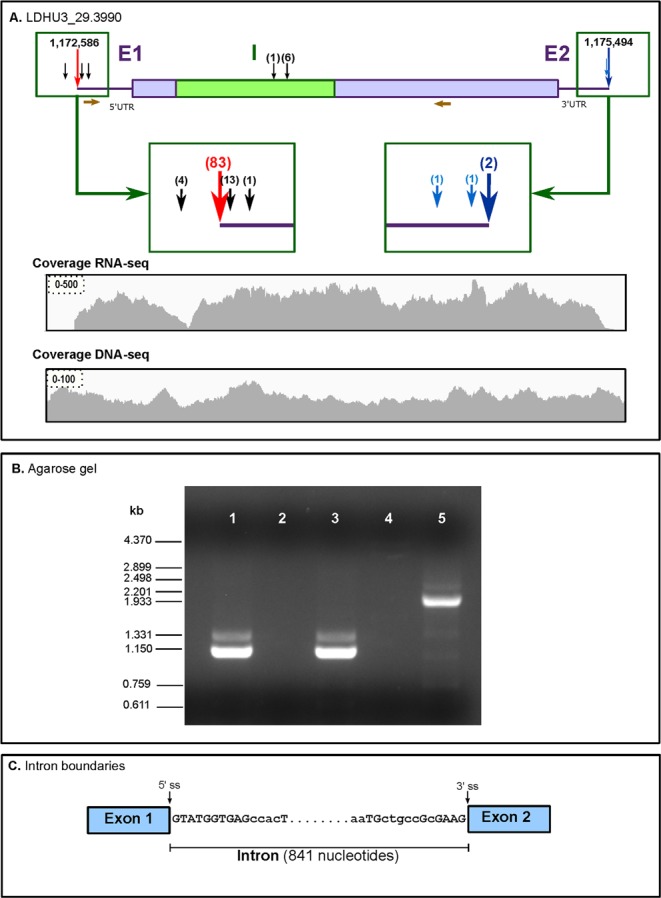
Processing by *cis-*splicing of the transcript encoding for the poly-A polymerase in *L*. *donovani*. Panel A, gene model for LDHU3_29.3990; E1 and E2, exons; I, intron. The red arrow indicates the position of the main SAS and the alternative SASs are indicated by black arrows (the number of RNA-seq mapped to each SAS is shown in parentheses). Blue arrows point to the poly-A addition sites. SASs mapped in the intron sequence are indicated by arrows above it. The position of the primers used for the PCR amplification are shown (maroon arrows; forward: 5′-GCGAGTTTCT GAAGTGCTGC-3′; reverse: 5′-TTCAGCACTG GGAACAGGTC-3′). The distribution (coverage) of Illumina reads along the region in study obtained after mapping of either RNA-derived reads (coverage RNA-seq) or DNA-derived reads (coverage DNA-seq) are also shown. Panel B, electrophoresis of PCR products on a 1% agarose gel; lanes 1 and 3, PCR amplification using cDNA derived from *L*. *donovani* total RNA and using for retrotranscription either SuperScript III (lane 1) or ThermoScript (lane 3) retrotranscriptases; lanes 2 and 4, PCR amplification from *L*. *donovani* total RNA (without previous retrotranscription step); lane 5, PCR amplification from *L*. *donovani* total DNA. Relative migration and size of molecular weight markers (Φ29 DNA digested with *Hin*dIII) are shown on the left. Uncropped gel shown in Supplementary Information Fig. S12. Panel C, schematic representation of the exon-intron junctions as determined after sequencing of the RT-PCR amplicon. Conserved nucleotides (upper case) in the equivalent intron existing in the gene coding for poly-A polymerase in *T*. *brucei*^86^. The positions of 5′ and 3′ splice sites (5′ ss and 3′ ss, respectively) are indicated.

During gene annotation, LDHU3_29.3990 gene, coding for a putative poly(A) polymerase (PAP), was initially annotated as pseudogene due to the presence of premature stop codons. Nevertheless, the homologous genes in *T*. *brucei* and *T*. *cruzi* are known to represent an exception to the general rule that genes in trypanosomatids are intron-less. Mair and co-workers^86^, by comparison of PAP genes in *T*. *brucei* and *T*. *cruzi*, suggested that an intronic sequence of 653 and 302 nt, respectively, would be present. Afterwards, these authors demonstrated experimentally that this intron is removed by *cis-*splicing in *T*. *brucei*, yielding a translatable mRNA expressing the complete PAP protein^86^. In both organisms, the intron occurs at identical positions within the CDS and obey the GT/AG rule of *cis*-splicing introns. In order to determine whether the orthologous gene in *L*. *donovani* (i.e., LDHU3_29.3990), after transcription, is also processed by *cis-*splicing, we firstly analysed the distribution of RNA-seq reads on the genomic region expanding the gene (Fig. 4A). The presence of a sudden drop in coverage around the genomic coordinate 1,173,206 caught our attention (this position is covered only by 24 reads, whereas the rest of the transcript is covered by around 400 reads). This valley was not observed when DNA-seq reads were mapped, indicating that it is not a region with special complexity for sequencing. Thus, looking for a possible *cis-*splicing processing of the LDHU3_29.3990 gene, the genomic sequence was analysed to define the putative exonic sequences, based on the amino acids encoded in the three direct reading frames. Interestingly, two putative exons accounting for the complete protein sequence were depicted. Based on this picture, a pair of oligonucleotides were designed, each one in a different exonic sequence, and used for PCR amplification from oligo d(T)-primed cDNA. An amplification product of around 1,100-nt was observed when the cDNA was used as template, whereas the expected PCR product of 1,949-nt in length was observed when genomic DNA was used as template (Fig. 4B). After cloning the 1,100-nt product, three independent clones were sequenced. In all three, the cloned sequence was identical and its length was 1,108-nt. The analysis of the sequence allowed us to determine that the LDHU3_29.3990 gene had an intron of 841-nt in length that would be removed post-transcriptionally by *cis-*splicing. Figure 4C shows a schematic representation of the intronic region of the LDHU3_29.3990 gene. It is remarkable the high sequence conservation existing between *T*. *brucei* (and *T*. *cruzi* too) and *L*. *donovani* intron sequences at the 5′-end. The first ten nucleotides are identical, whereas at the 3′-end only four nucleotides are conserved (Fig. 4C). In *T*. *brucei*, it was demonstrated that punctual substitutions of any of the 10 conserved nucleotides at 5′-end of the intron led to a total inhibition of the intron removal^86^. The authors suggested that this sequence conservation is being dictated by the sequence complementarity existing between the exon1-intron boundary and the U1 snRNA, putatively involved in the splicing process.

The coverage of RNA-seq reads over the LDHU3_29.3990 genomic locus did not show a clear decline along the intronic region, suggesting a non-efficient *cis-*splicing and intron removal, at least in the promastigote stage. The existence of a low read depth in position close to the 5′ splice site (Fig. 4A) would be an indication that pre-mRNA is being cut at that position, but intron removal from exon 2 would be occurring inefficiently. Also, it should be noted the existence of two SAS in the intronic sequence, just upstream from the second exon. Mair *et al*.^86^ also found that some RNA molecules derived from the orthologue gene in *T*. *brucei* are processed by *trans*-splicing at a cryptic site located within the intron. It would be interesting to know whether this shortened mRNA is translated and, if so, whether a functional protein is generated.

There is another documented case of *cis*-splicing in trypanosomatids. The *Trypanosoma brucei* gene Tb927.8.1510, encoding an ATP-dependent RNA helicase (DBP2B), has been found to be processed by *cis*-splicing^87^. Similarly, the orthologous gene in *L*. *major* (LmjF.07.0340) was annotated as *cis*-spliced gene by Peter Myler at TriTrypDB. To address the possibility that the orthologous gene in *L*. *donovani* (LDHU3_07.0430) is also processed by *cis*-splicing, a search for splice junctions among the RNA-seq reads was performed using the Tophat2 tool aligner^88^. The results were clear, a significant number of RNA-seq reads covering the exon-junction site were identified; this information allowed us to precisely map the position of the two exons in the genomic sequence and to determine that the gene is split by a 4,470-bp long intron (see Fig. S11 in Supplementary Information File 1).

### Determination of RNA levels from RNA-seq data

RNA-seq data produce digital counts of transcript abundance, allowing to quantify transcript levels in a straightforward manner. Nevertheless, basic for an accurate determination of expression levels is the availability of appropriate gene models, as determined in this work for *L*. *donovani*. To measure relative expression levels of the transcripts, we used RNA-seq reads derived from three biological replicates of *L*. *donovani* promastigotes to determine FPKM values for the 10,893 gene models established in this study. Table 5 lists the 50 most abundant transcripts in logarithmic phase promastigotes. It is not surprising that the list is headed by two histone transcripts and that another seven histone genes were also present among the 50 most abundant transcripts. Similarly, the high cellular requirements of tubulin agree with the high expression levels determined for seven different alpha-tubulin genes. Also, transcripts coding for 13 ribosomal proteins were found among the top 50 transcripts. In agreement with previous studies in other *Leishmania* species^14,89^, HSP70 (type-II gene) and KMP11 transcripts were also found among the most abundant transcripts. Many of the most expressed genes in *L*. *donovani* were also identified among the most highly expressed genes in the *L*. *tropica* promastigote stage^83^. Additionally, among the highly expressed genes, four lacking of CDS annotation (LDHU3.10.T1390, LDHU3.27.T1760, LDHU3.30.T1180, and LDHU3.36.T5090) were present. Given their abundance, further analysis should be performed in order to determine whether they have protein-coding capacity or definitively represent non-coding RNAs (ncRNAs).

**Table 5:** The 50 most abundant transcripts in *L*. *donovani* (HU3 strain) promastigotes.

Transcript	Protein product	FPKM (± SD)
LDHU3_09.T1660	histone H2B	3677.33 (± 38.61)
LDHU3_06.T0020	histone H4	3642.62 (± 45.66)
LDHU3_27.T1700	hypothetical protein	3388.42 (± 41.35)
LDHU3_29.T2670	histone H2A	2428.46 (± 38.17)
LDHU3_31.T1470	hypothetical protein, conserved	2317.52 (± 21.57)
LDHU3_13.T0480	Alba	2160.44 (± 29.93)
LDHU3_27.T1760	Non annotated CDS	2045.59 (± 36.76)
LDHU3_15.T0010	histone H4	1902.71 (± 37.44)
LDHU3_17.T1420	META domain containing protein	1710.65 (± 17.22)
LDHU3_13.T0630	40 S ribosomal protein S12 putative	1668.54 (± 21.42)
LDHU3_27.T0170	WW/Zinc finger domain containing protein	1611.85 (± 21.22)
LDHU3_10.T1400	histone H3	1605.27 (± 26.92)
LDHU3_28.T3970	HSP70 (gene HSP70-II)	1565.85 (± 12.38)
LDHU3_15.T1550	nucleoside transporter 1	1503.34 (± 11.69)
LDHU3_30.T0880	40 S ribosomal protein S30	1473.70 (± 64.82)
LDHU3_19.T0030	histone H2B	1470.15 (± 23.58)
LDHU3_23.T2590	Nucleoside 2-deoxyribosyltransferase	1450.04 (± 24.23)
LDHU3_36.T5090	Non annotated CDS	1450.04 (± 28.18)
LDHU3_36.T5100	EF-hand domain containing protein	1447.41 (± 26.13)
LDHU3_26.T2920	ribosomal protein L38	1275.35 (± 28.52)
LDHU3_13.T0400	alpha tubulin	1258.35 (± 15.09)
LDHU3_13.T0370	alpha tubulin	1256.55 (± 15.07)
LDHU3_13.T0360	alpha tubulin	1238.15 (± 14.97)
LDHU3_30.T1180	No annotated CDS	1223.66 (± 16.36)
LDHU3_19.T0040	histone H2B	1207.16 (± 28.86)
LDHU3_13.T0350	alpha tubulin	1206.51 (± 14.76)
LDHU3_36.T1660	fructose-16-bisphosphate aldolase	1154.03 (± 15.42)
LDHU3_33.T4780	40S ribosomal protein S13	1122.95 (± 28.51)
LDHU3_33.T1080	60S ribosomal protein L6	1111.59 (± 23.27)
LDHU3_15.T1290	40S ribosomal protein S3	1107.49 (± 18.96)
LDHU3_24.T2850	60S ribosomal protein L12	1082.74 (± 24.63)
LDHU3_21.T1370	histone H2A	1074.84 (± 24.22)
LDHU3_10.T1390	No annotated CDS	1063.97 (± 24.84)
LDHU3_20.T1990	40S ribosomal protein S11	1056.10 (± 21.5)
LDHU3_35.T0570	40S ribosomal protein S3A	1035.42 (± 18.85)
LDHU3_35.T2890	kinetoplastid membrane protein-11	1033.21 (± 15.14)
LDHU3_24.T2590	60S ribosomal protein L26	1030.16 (± 21.2)
LDHU3_13.T0340	alpha tubulin	1010.16 (± 12.49)
LDHU3_19.T0050	histone H2B	1007.14 (± 26.18)
LDHU3_21.T2650	Tubulin C domain containing protein	1004.59 (± 11.1)
LDHU3_08.T0720	Amastin surface glycoprotein	1002.89 (± 9.58)
LDHU3_26.T3080	60S ribosomal protein L35	995.32 (± 18.85)
LDHU3_36.T2660	inosine-guanosine transporter	995.13 (± 10.33)
LDHU3_32.T3530	L-Lysine transport protein	991.13 (± 8.017)
LDHU3_32.T0990	RNA binding protein	986.09 (± 14.73)
LDHU3_33.T1490	hypothetical protein conserved	970.46 (± 19.07)
LDHU3_13.T0390	alpha tubulin	963.41 (± 11.52)
LDHU3_16.T1490	60S ribosomal protein L39	951.77 (± 21.3)
LDHU3_35.T0740	60S ribosomal protein L18a	949.84 (± 16.3)
LDHU3_13.T0380	alpha tubulin	943.59 (± 11.4)

In a previous work from our group, we determined the 50 most abundant transcripts in *L*. *major* promastigotes^14^. A comparison between both studies evidenced, apart from the structural abundant proteins indicated above, a similar high expression of the following transcripts: LDHU3_31.T1470 (coding for a conserved hypothetical protein), LDHU3_13.T0480 (Alba protein), LDHU3_15.T1550 (nucleoside transporter 1), LDHU3_36.T5100 (EF-hand domain containing protein), LDHU3_08.T0720 (amastin), and LDHU3_36.T2660 (inosine-guanosine transporter).

## Conclusions

As proved recently with the *L*. *infantum* genome assembly^22^, combination of the Illumina sequencing accuracy and the long sequence reads obtained by the PacBio platform has resulted adequate to obtain an almost complete assembly of the *L*. *donovani* genome.

The availability of a well-assembled genome is pivotal for undertaking global genomics, transcriptomics and proteomics studies with confidence. Here, based on the assembled genome, we have delineated the poly-A+ transcriptome, consisting in 10,893 transcripts (see Supplementary File 2 for the complete list). Remarkably, 2,410 are transcripts that do not contain annotated ORFs. Furthermore, transcript location served to correct several hundreds of miss-annotated ORFs, which are usually predicted by bioinformatics tools (Companion, in this study). As reported for *L*. *major*^14^ and for *L*. *mexicana*^40^, the transcriptome of *L*. *donovani* also shows a remarkable heterogeneity in the sites used for the mini-exon (SL) and polyadenylation addition. Thus, alternative *trans*-splicing and polyadenylation may represent an additional and relevant points for controlling gene expression in *Leishmania*.

Analysis of gene expression, undertaken either individually or genome wide, requires accurate gene models in which, in addition to the ORF, the UTRs must be precisely delimitated. In fact, most of the regulatory elements involved in post-transcriptional mechanisms of gene expression accumulate in UTRs^90^, and regulation of gene expression in *Leishmania* and other trypanosomatids is essentially post-transcriptional^9,15^. Additionally, the availability of a complete *L*. *donovani* transcriptome will allow to carry out transcriptome profiling associated with drug resistance in this species without the necessity of using data derived from other *Leishmania* species^59,91^.

As shown previously for other *Leishmania* species^80^, the *L*. *donovani* genome is not very different to the genome of other species regarding the number of different proteins that are encoded. Thus, only fifteen out of the 8,405 genes annotated as protein-coding lacked orthologues in the *L*. *major* genome. This number of genes may seem too low to explain the different pathologies that these species produce in humans: *L*. *donovani* causes fatal viscerocutaneous affectations, whereas *L*. *major* infection generates self-curing cutaneous lesions. However, large differences were found regarding the number of distinct pseudogenes and the gene copy number variations existing between both species. Thus, in agreement with other comparative genomics studies^78,83^, we suggest that pseudogene formation and variations in gene copy numbers may be greatly contributing to the genetic basis for disease tropism.

Finally, we have demonstrated that the transcripts coding for the poly(A) polymerase (PAP) and for an ATP-dependent RNA helicase in *L*. *donovani* are processed by *cis-*splicing, following a similar pattern of processing to that described for the orthologous genes in other trypanosomatids. These results suggest an ancient acquisition of these particular events of *cis*-splicing in the evolutionary line of these protists, where *cis-*splicing is considered essentially absent. Surely, a relevant physiological function could be behind the conservation of this, apparently fossilized, molecular mechanism.

## Supplementary information

Supplementary Information

Dataset 1

Dataset 2

## Data Availability

Both genomic and transcriptomic raw data have been deposited in The European Nucleotide Archive (ENA; http://www.ebi.ac.uk/ena/). The assembled genome and transcriptome sequences together with annotations files were uploaded under the Study accession number PRJEB23341 and Study unique name: ena-STUDY-CBMSO-06-11-2017-13:36:27:181–100. Additionally, Fasta files with the genome sequence and transcriptome are downloadable at the Leish-ESP web site (http://leish-esp.cbm.uam.es/).
