# Supplementary material for: Complete assembly of the Leishmania donovani (HU3 strain) genome and transcriptome annotation
Source: Sci Rep. 2019 Apr 16;9:6127. doi: 10.1038/s41598-019-42511-4 (PMC6467909; doi:10.1038/s41598-019-42511-4)
Supplement: Supplementary file 1 — Supplementary Information [file 41598_2019_42511_MOESM1_ESM.pdf]

SUPPLEMENTARY INFORMATION

Complete assembly of the *Leishmania donovani* (HU3 strain) genome and transcriptome annotation

Esther Camacho, Sandra González-de la Fuente, Alberto Rastrojo, Ramón Peiró-Pastor, Jose Carlos Solana, Laura Tabera, Francisco Gamarro, Fernando Carrasco-Ramiro, Jose M. Requena, Begoña Aguado

Legends to Figures S1-S10. Schematic illustration of chromosomal reorganizations found between the *Leishmania donovani* genome (HU3 strain) assembled in this work and the current reference genome (*L. donovani* BPK strain; TriTrypDB). Pairwise alignments between the HU3 (top) and BPK (bottom) genomes were generated using Mauve genome alignment tool with default settings (Darling et al., 2010). This tool uses different colors to represent synteny blocks. Sections located underneath the x-axis show inversion events. [Darling, A. E., Mau, B. & Perna, N. T. (2010). progressiveMauve: multiple genome alignment with gene gain, loss and rearrangement. PLoS One 5, e11147. DOI:10.1371/journal.pone.0011147]

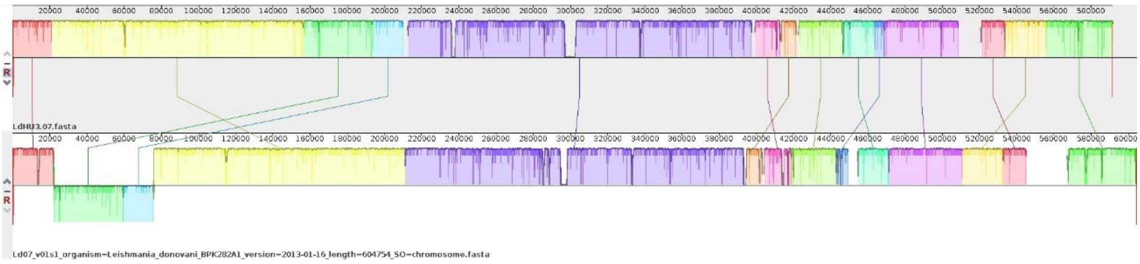

Figure S1. Reorganizations found in chromosome 7.

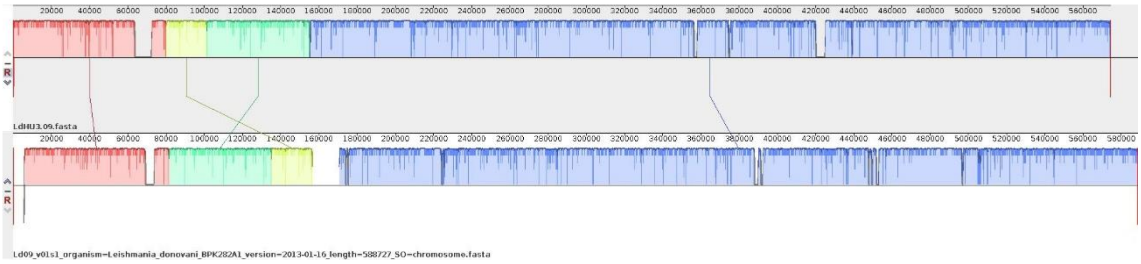

Figure S2. Reorganizations found in chromosome 9.

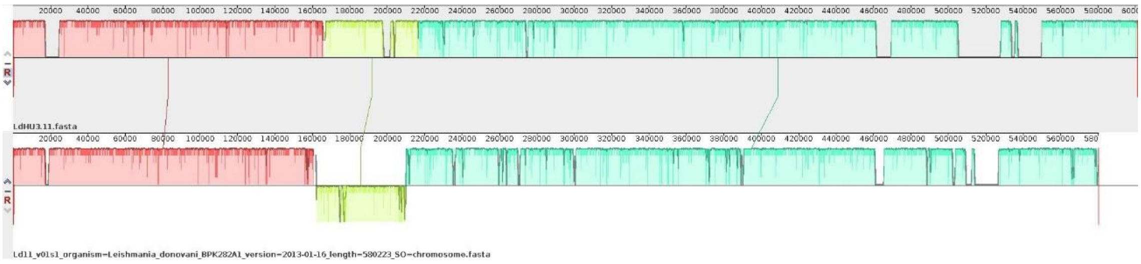

Figure S3. Reorganizations found in chromosome 11.

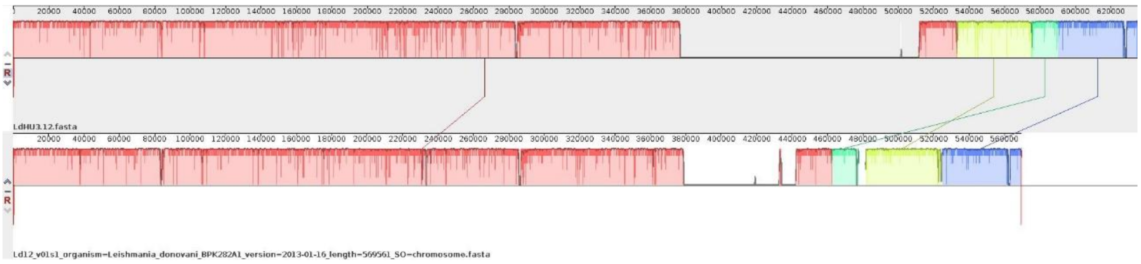

Figure S4. Reorganizations found in chromosome 12.

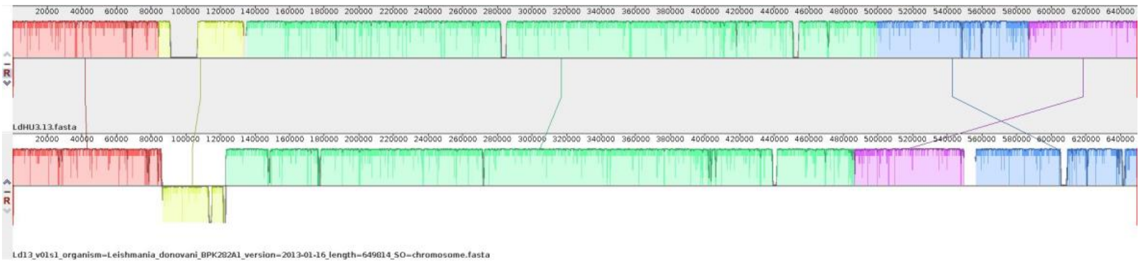

Figure S5. Reorganizations found in chromosome 13.

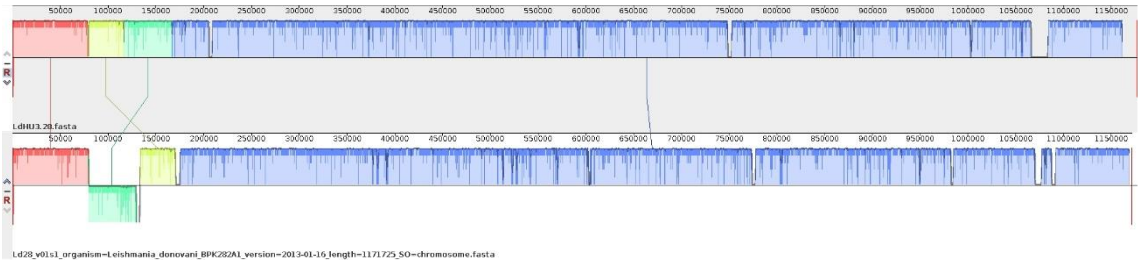

Figure S6. Reorganizations found in chromosome 28.

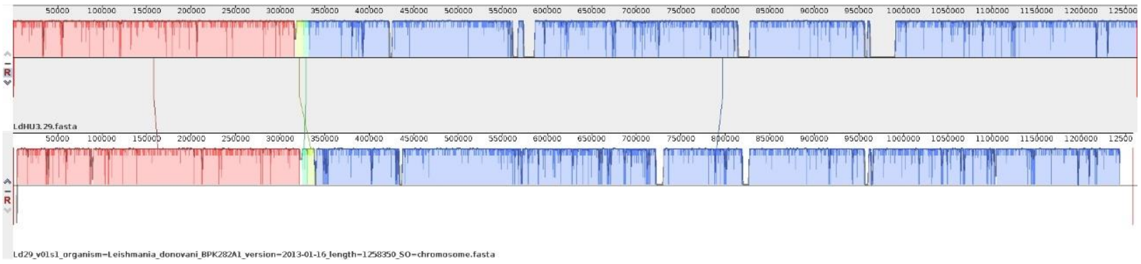

Figure S7. Reorganizations found in chromosome 29.

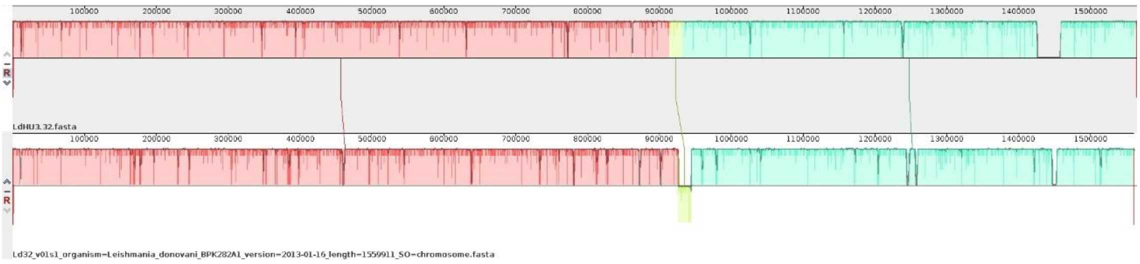

Figure S8. Reorganizations found in chromosome 32.

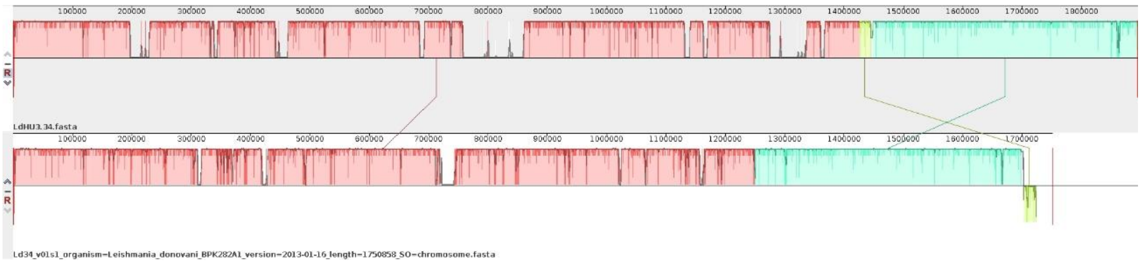

Figure S9. Reorganizations found in chromosome 34.

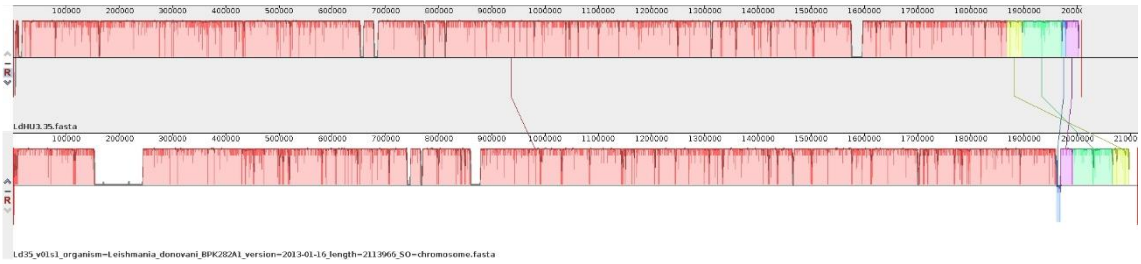

Figure S10. Reorganizations found in chromosome 35.

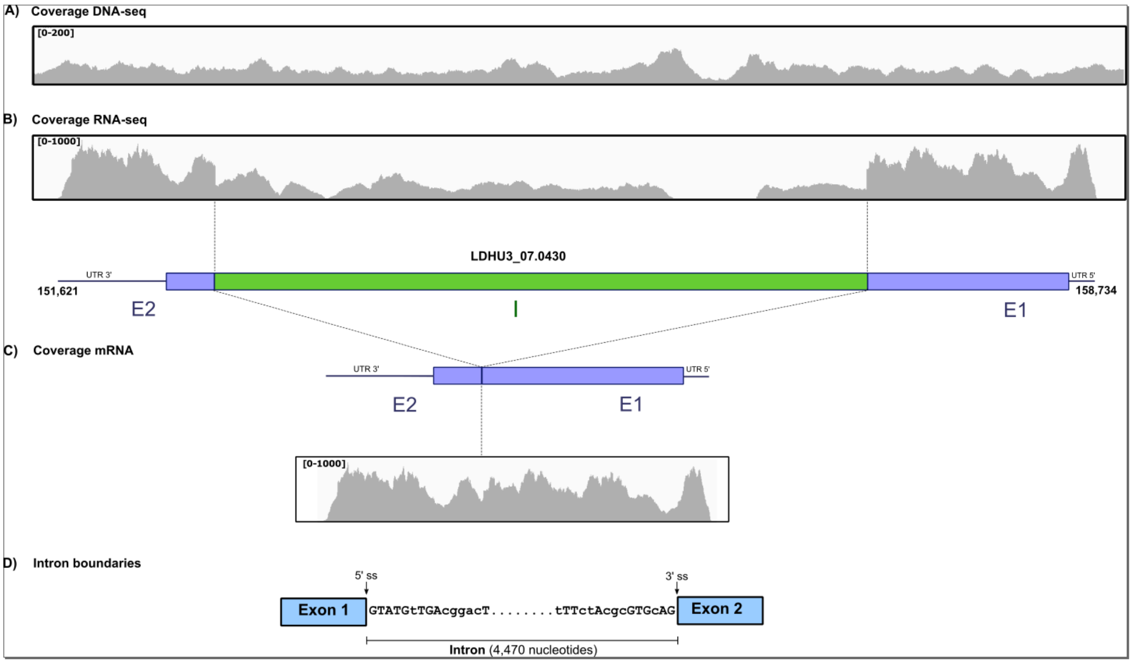

Figure S11. Processing by cis-splicing of the transcript encoding for the ATP-dependent RNA helicase in *L. donovani*. Panel A, distribution (coverage) of Illumina reads along the region of study, obtained after mapping of DNA-derived reads (coverage DNA-seq). Panel B, distribution (coverage) of Illumina reads along the region of study, obtained after mapping of RNA-derived reads (coverage RNA-seq). Schematic representation of the gene model LDHU3_07.0430, exons (purple) and intron (green). Panel C, distribution (coverage) of Illumina reads along the emulated mRNA (gene model without intron), obtained after mapping of RNA-derived reads into the region. Panel D, schematic representation of the exon-intron junctions as determined after the analysis of the predicted genome junctions. Conserved nucleotides (upper case) in the equivalent intron existing in the gene coding for poly-A polymerase (see Fig. 4). The positions of 5’ and 3’ splice sites (5’ ss and 3’ ss, respectively) are indicated.

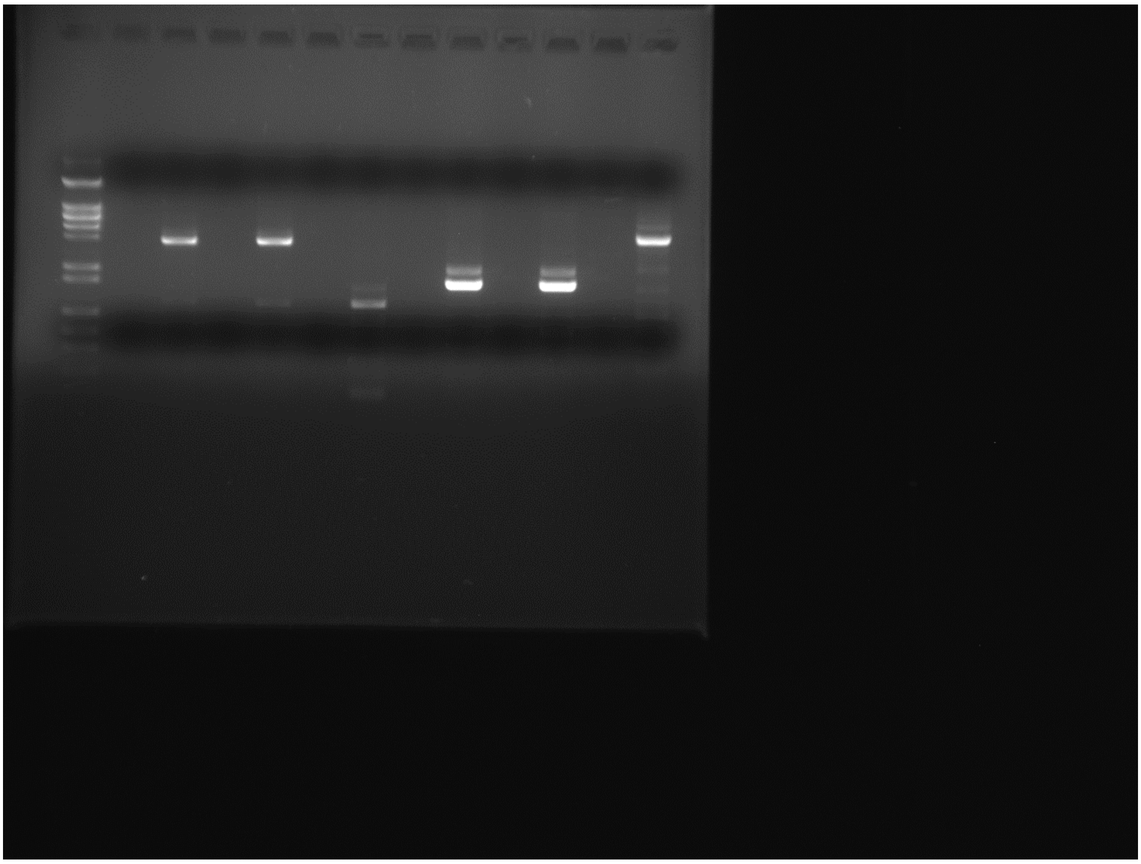

Figure S12.
Image of the complete agarose gel in which the RT-PCR samples, shown in panel B of figure 4, were loaded. In figure 4 the five lines located on the right-hand of the gel are shown. The lane on the left contains Φ29 DNA digested with *Hin*dIII that was used as a molecular weight marker.
